# The neuroepithelial basement membrane serves as a boundary and a substrate for neuron migration in the zebrafish hindbrain

**DOI:** 10.1186/1749-8104-5-9

**Published:** 2010-03-29

**Authors:** Paul K Grant, Cecilia B Moens

**Affiliations:** 1 HHMI and Division of Basic Science, Fred Hutchinson Cancer Research Center, 1100 Fairview Ave N, Seattle, WA 98109-1024, USA; 2 Molecular and Cellular Biology Program, University of Washington, Seattle, WA 98195-7470, USA

## Abstract

**Background:**

The facial branchiomotor neurons of cranial nerve VII undergo a stereotyped tangential migration in the zebrafish hindbrain that provides an ideal system for examining the complex interactions between neurons and their environment that result in directed migration. Several studies have shown the importance of the planar cell polarity pathway in facial branchiomotor neuron migration but the role of apical-basal polarity has not been determined. Here we examine the role of the PAR-aPKC complex in forming the basal structures that guide facial branchiomotor neurons on an appropriate migratory path.

**Results:**

High resolution timelapse imaging reveals that facial branchiomotor neurons begin their migration by moving slowly ventrally and posteriorly with their centrosomes oriented medially and then, upon contact with the Laminin-containing basement membrane at the rhombomere 4-rhombomere 5 boundary, speed up and reorient their centrosomes on the anterior-posterior axis. Disruption of the PAR-aPKC complex members aPKCλ, aPKCζ, and Pard6gb results in an ectopic ventral migration in which facial branchiomotor neurons escape from the hindbrain through holes in the Laminin-containing basement membrane. Mosaic analysis reveals that the requirement for aPKC is cell-nonautonomous, indicating that it is likely required in the surrounding polarized neuroepithelium rather than in facial motor neurons themselves. Ventral facial motor neuron ectopia can be phenocopied by mutation of *lamininα1*, suggesting that it is defects in maintenance of the laminin-containing basement membrane that are the likely cause of ventral mismigration in aPKCλ+ζ double morphants.

**Conclusions:**

Our results suggest that the laminin-containing ventral basement membrane, dependent on the activity of the PAR-aPKC complex in the hindbrain neuroepithelium, is both a substrate for migration and a boundary that constrains facial branchiomotor neurons to the appropriate migratory path.

## Background

Spatial information along the anterior-posterior and dorsal-ventral axes is used to specify neuronal identity during vertebrate brain development. The position at which a neuron's fate is specified, however, frequently does not correspond to the position at which it must perform its function. Instead, neurons frequently migrate from their place of birth to their final functional position. This process of neuronal migration involves a tightly choreographed interaction between a migrating cell and the complex environment through which it is migrating, requiring the organism to lay down the appropriate cues, substrates, and boundaries that guide migration at the appropriate time during development [1].

The tangential migration of the facial branchiomotor neurons (FBMNs) of cranial nerve VII in the zebrafish hindbrain provides an ideal system for examining the complex interactions between migrating neurons and their environment. FBMNs are continuously differentiating in rhombomere 4 (r4) starting at 16 hours post-fertilization (hpf). They migrate posteriorly to r6 and r7, the migration lasting 4 to 6 hours for each FBMN, and the entire migration of about 100 to 150 μm is concluded by 48 hpf [2]. During this period the zebrafish hindbrain constitutes a richly patterned and dynamic environment. Early in migration, the hindbrain is a pseudostratified neuroepithelium composed of neural progenitor cells that, over the course of the time that migration progresses, undergo neurogenesis and gliogenesis [3].

The early hindbrain neuroepithelium is polarized both apico-basally and within the plane of the epithelium. This latter form of polarity, known as planar cell polarity (PCP), has been shown to be required for FBMN migration. Mutations in the PCP genes *vangl2 *[4,5], *prickle1b *[6], *frizzled3a*, and *celsr2 *[7] all result in a failure of FBMNs to migrate out of r4. It has been proposed that PCP proteins function by excluding FBMNs from the more dorsal neuroepithelium and keeping them on their appropriate migratory pathway [7,8]. The role of apical-basal polarity in FBMN migration, however, has not been assessed. FBMNs undergo the majority of their migration apposed to the ventral pial surface, which corresponds to the basal domain of the neuroepithelial progenitor cells. The apical domain of these progenitors is located at the midline where the lumen is forming during this stage of development [9].

A key regulator of apical-basal polarity in many systems is the PAR-aPKC complex, composed of two scaffold proteins, PAR-3 and PAR-6, and a serine/threonine kinase, aPKC [10]. In zebrafish there is one PAR-3 gene, *pard3*, two aPKC genes,*aPKCλ *and *aPKCζ*, and four PAR-6 genes, *pard6a*, *pard6b*, *pard6ga*, and *pard6gb *[10,11]. The PAR-aPKC complex functions to polarize epithelial cells by establishing apical membrane domains and promoting the formation of apical structures such as tight junctions [10]. Members of the PAR-aPKC complex have also been shown to have a role in polarizing migrating cells by reorienting the centrosome in migrating wound-edge astrocytes [12], and controlling the saltatory migration of cortical neurons along their glial guides [13].

The PAR-aPKC complex also functions indirectly to maintain basal structures of epithelia by maintaining the basolateral localization of proteins such as PAR-1 and the Lgl-Dlg-Scrib complex. The basement membrane is a sheetlike extracellular matrix composed of a network of Laminin and type IV Collagen crosslinked and decorated with various other protein components. It anchors the basal domain of epithelia and functions in their polarization [14]. Basement membrane components are secreted by epithelial cells and organized into basal sheets by basally located Dystroglycan complex that anchors and allows for the polymerization of Laminin [15]. The basal localization of the Dystroglycan complex requires basally-localized Par1, which in turn requires apical localization of PAR-3-PAR-6-aPKC [16].

Here we show that FBMN migration requires the basal structures produced by a properly polarized neuroepithelium. We observe that FBMNs undergo a change in their migratory behavior upon contact with the ventral Laminin-containing basement membrane. Depletion of PAR-aPKC complex members or of Laminin itself results in defects in the basement membrane that allow FBMNs to mismigrate ventrally, escaping the hindbrain entirely. This suggests that the ventral Laminin-containing basement membrane is both a substrate for FBMN migration and a barrier that constrains that migration to its appropriate trajectory.

## Results

### FBMNs undergo a change in velocity and centrosome orientation upon contact with the ventral basement membrane

**Figure 1:**
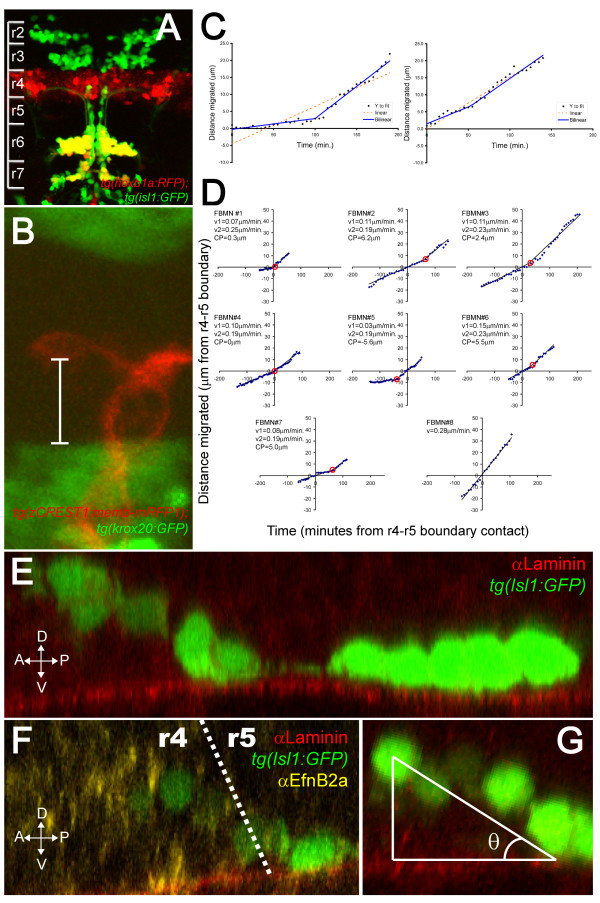
**FBMNs undergo a change in velocity upon contact with the Laminin-containing ventral basement membrane**. **(A) **A maximum intensity projection of a confocal Z-stack of a *tg(hoxb1aBAC:mRFP1); tg(isl1:GFP) *double transgenic embryo at 48 hpf reveals that most Isl1:GFP positive neurons in both r6 and r7 are also hoxb1a:RFP positive, indicating that they were born in r4. **(B) **A single XY section of a *tg(zCREST1:memb-mRFP1);tg(krox20BAC:GFP) *double transgenic embryo at approximately 24 hpf showing the measurement from the posteriormost extent of the FBMN cell body to the r4-r5 boundary (white bar). **(C) **Plots of the extent of migration of single FBMNs versus time elapsed generated from timelapse movies of a *tg(zCREST1:memb-mRFP1);tg(krox20BAC:GFP) *double transgenic embryo starting at approximately 24 hpf. In the left graph, points are best fit by a bilinear model rather than a single line, indicating that the FBMN undergoes a single change in velocity while the right graph is equally well fit by either model, indicating a constant velocity. **(D) **Plots of extent of migration versus time re-centered so that the origin of the graph corresponds to the point of contact with the r4-r5 boundary (negative timepoints and distances indicate migration through r4 while positive timepoints and distances indicate migration through r5). FBMN#1 to FBMN#7 are those best fit by a bilinear model while FBMN#8 is an example of one best fit by a single line. CP, changepoint, the point at which the slope changes in the bilinear model (red circle); v1, initial slope; v2, slope after changepoint. **(E-G) **Reconstructed YZ sections through 24-hpf isl1:GFP embryos stained for Laminin (E) or Laminin (red) and α Efnb2a (yellow) (F) reveal that FBMNs undergo a ventral migration in r4 and come into close apposition with the basement membrane just after crossing the r4-r5 boundary, after which point their migration is purely posterior. (G) The angle of the ventroposterior early migration with respect to the basement membrane (θ) is measured by defining a right triangle with vertices at the middle of the anteriormost cell, the point of contact with the basement membrane and the basement membrane as one of the legs.

Facial branchiomotor neurons are born in hindbrain r4, first becoming detectable by Isl1:GFP expression at 16 hpf, and migrate posteriorly to r6 and r7 by 48 hpf. This is apparent in *tg(hoxb1aBAC:RFP); tg(isl1:GFP) *double transgenic fish, where cells born in r4 express red fluorescent protein (RFP) and branchiomotor neurons (including FBMNs) express GFP. In these double transgenics, most FBMNs in r6 and r7 are GFP- and RFP-positive, indicating their r4 origins (Figure 1A).

In order to better visualize the migration of FBMNs in live embryos, we created a transgenic line in which a membrane-targeted monomeric RFP [17] is expressed in branchiomotor neurons under the control of the minimal *islet1 *enhancer, zCREST1 [18]. This transgenic line, *tg(zCREST1:memb-mRFP1)*, allows for timelapse movies that show the cellular behaviors of migrating FBMNs in greater detail than has previously been possible. New FBMNs immediately begin their posterior migration, moving in an amoeboid manner without a prominent leading process. FBMNs appear to rappel down the hindbrain, playing out their axons behind them. This axon tract seems to serve as a scaffold, allowing later born neurons to migrate in close apposition with the axons of earlier born neurons and for FBMNs to crawl over each other as they migrate (Additional files 1, 2 and 3).

Crossing the *tg(zCREST1:memb-mRFP1) *transgenic line with a *tg(krox20BAC:GFP) *transgenic line that expresses GFP in r3 and r5 allowed us to measure the posterior migratory velocity with respect to the r4-r5 boundary (Figure 1B; Additional file 1).

We measured the extent of individual FBMN posterior migration over time for 14 FBMNs from 12 different embryos, with the onset of imaging occurring between 18 and 28 hpf. When we pooled these data and compared the average velocity for all observed FBMNs in r4 (0.11 ± 0.01 μm/minute) versus their average velocity in r5 (0.17 ± 0.01 μm/minute), we observed a statistically significant increase in velocity in r5 (*t*-test; *P *< 0.01). In order to determine the nature of this increase in velocity, we plotted the extent of posterior migration versus time and compared the fit of various models to the data. We found that for 7 of the 14 cells, the data was best fit by a bilinear model [19], indicating a single change in velocity (Figure 1C, left graph), while for the other 7 cells, the data was equally well fit by a bilinear model or by a single line, indicating a constant velocity (Figure 1C, right graph). In no cases was an exponential model superior to the bilinear model (data not shown). When we re-centered these plots so that the origin of the graph corresponded to the point of contact with the r4-r5 boundary, it became apparent that the change in velocity occurred within 7 μm (about one cell diameter) from the r4-r5 boundary (Figure 1D, FBMN#1 to FBMN#7).

We wondered what was occurring near the r4-r5 boundary that might explain this change in velocity. We performed a three-dimensional analysis of Isl1:GFP transgenic embryos [20] stained with anti-Laminin antibody. This analysis revealed that FBMNs begin their migration by moving ventrally as well as posteriorly until they reach the ventral Laminin-containing basement membrane. After this, migration is purely posterior (Figure 1E). To determine where on the anterior-posterior axis FBMNs reach the basement membrane, we immunostained *tg(isl1:GFP) *embryos for Laminin and EfnB2a, which is expressed in rhombomere 4 (r4). This staining revealed that the point of contact with the ventral basement membrane occurs at approximately the r4-r5 boundary (Figure 1F). The slow phase of migration, then, correlates with the ventro-posterior movement that occurs before neurons reach the basement membrane while the fast phase correlates with purely posterior movement that occurs in close apposition to the basement membrane.

The velocity measurement we made is the posterior component of a velocity vector that points ventro-posteriorly during the slow phase of migration and then points posteriorly after cells have reached the ventral basement membrane. As such, the change in posterior velocity might be explained simply by this change in direction. To correct for the ventral component of migration, we calculated the average angle (θ) between the line of ventrally migrating neurons and the ventral basement membrane (Figure 1G) and multiplied the initial velocity measurement by the correction factor of 1/cosθ to arrive at the magnitude of the ventro-posterior velocity vector. Even after this correction factor is applied, FBMNs migrate with an average velocity of 0.11 ± 0.01 μm/minute during the ventral-directed phase while they migrate at a significantly different velocity of 0.21 ± 0.01 μm/minute during the posterior phase (*t*-test; *P *< 0.0005). This indicates that apposition with the basement membrane correlates with an increased rate of migration. FBMNs that migrated at a constant velocity (for example, Figure 1D, FBMN#8) did so with an average velocity of 0.18 μm/minute, so we speculate that most of these FBMNs are migrating in the fast, posterior phase of migration, suggesting that they may have contacted the basement membrane before we began recording.

**Figure 2:**
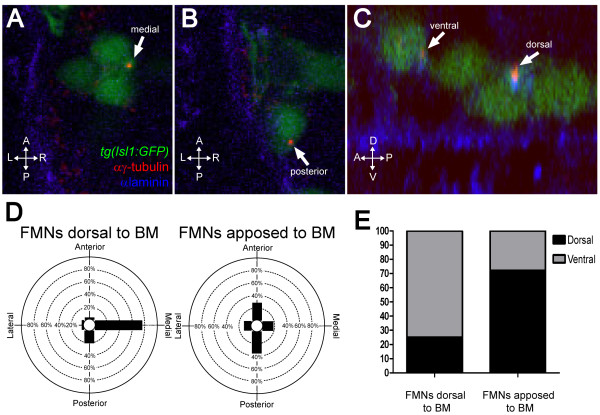
**FBMNs undergo a change in centrosome orientation upon contact with the Laminin-containing ventral basement membrane**. **(A, B) **Single XY sections of 24-hpf embryos stained for Laminin (blue) and γ-tubulin (red) reveal that FBMNs that have not yet contacted the basement membrane (BM) (A) mostly orient their centrosomes medially (arrow) while FBMNs that are apposed to the BM (B) orient their centrosomes anteriorly or posteriorly (arrow). **(D) **Percentage of FBMNs dorsal to the BM whose centrosomes are found in the specified quadrant versus FBMNs apposed to the BM. **(C) **Reconstructed YZ sections of these same embryos reveal that centrosomes in FBMNs that are dorsal to the basement membrane are mostly in the ventral half of the cell while in FBMNs that are apposed to the basement membrane the centrosomes are mostly dorsal (arrows). **(E) **Percentage of FBMNs dorsal to the BM whose centrosomes are found in the dorsal or ventral halves versus FBMNs apposed to the BM.

In many migratory cell types, such as wound-edge astrocytes and fibroblasts [12,21] or migrating cortical neurons [22], the centrosome localizes between the nucleus and the leading edge, where it organizes the microtubule cytoskeleton in a polarized fashion. We asked whether the change we observed in migratory velocity might be associated with changes in polarity as assayed by centrosome position. We found that before contact with the ventral basement membrane, most centrosomes in FBMNs are found in the ventral half (75%; n = 36 FBMNs from 4 embryos) and in the medial quadrant (75%; n = 36 FBMNs from 4 embryos) of the cell (Figure 2A, D, E). Medial centrosome orientation is similar to that observed in the surrounding neuroepithelial progenitor cells whose centrosomes are found at the (apical) midline. In contrast, the majority of centrosomes of FBMNs that are apposed to the ventral basement membrane are in the dorsal half of the cell (72%; n = 36 FBMNs from 4 embryos) and in the anterior or posterior quadrants (28% and 44%, respectively; n = 36 FBMNs from 4 embryos) (Figure 2B, D, E). Chi-squared analysis reveals that the distribution of centrosomes in r5 is significantly different from that in r4 (*P *< 0.0001). Together, these data form a picture in which FBMNs begin their migration moving slowly ventro-posteriorly with their centrosomes pointing ventro-medially until they reach the basement membrane, where they cease ventral migration, reorient their centrosomes either dorso-anteriorly or dorso-posteriorly, and accelerate in the posterior direction.

### PAR-aPKC complex members aPKCλ, aPKCζ, and Pard6gb are required for proper FBMN migration

The above observations led us to speculate that there may be a requirement for proper cell polarity in the migrating FBMNs and/or in the surrounding neuroepithelial progenitors that lay down the basement membrane that appears to be a substrate for migration. Members of the PAR-aPKC complex have been shown to be responsible for both polarity in migrating cells (including centrosome reorientation [23]), and apical-basal polarity within epithelia [10]. We asked whether PAR-aPKC complex proteins have a role in FBMN migration.

**Figure 3:**
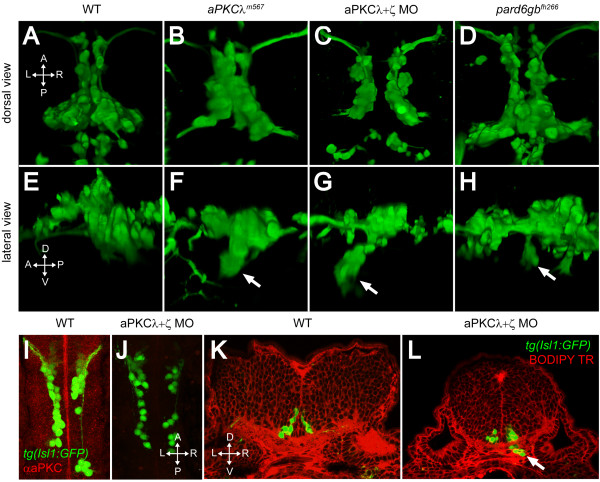
**PAR-aPKC complex disruption causes a ventral mismigration in which FBMNs escape the hindbrain ventrally**. **(A-H) **Three-dimensional reconstructions of confocal Z-stacks of the Isl1:GFP positive facial motor neurons at 48 hpf in wild-type (WT) (A, E), *aPKCλ*^*m*567 ^homozygous mutant (B, F), aPKCλ+ζ double morpholino knockdown (C, G), and *pard6gb*^*fh*266 ^homozygous mutant embryos (D, H) shown in dorsal (A-D) and lateral (E-H) views. In *aPKCλ*^*m*567^, aPKCλ+ζ double morpholino knockdown, and *pard6gb*^*fh*266 ^embryos (F, G, H), a subset of FBMNs mismigrates ventrally (arrows) forming ectopic ventral clusters. **(I, J) **Maximum intensity projections of confocal Z-stacks of 24-hpf WT (I) and aPKCλ+ζ MO (J) embryos stained with an antibody that recognizes both aPKCs reveals that double morpholino knockdown reduces aPKC to undetectable levels. **(K, L) **Vibratome coronal sections through 48-hpf WT (K) and aPKCλ+ζ MO (L) embryos at 48 hpf counterstained with BODIPY TR methylester dye that stains all membranes reveals that morphant FBMNs are found outside the hindbrain (arrow). In addition, the cross-sectional area of the aPKCλ+ζ MO hindbrain is reduced and the ventricle has not inflated.

In order to examine the role of aPKC in FBMN migration, we crossed the *heart and soul *(*has/aPKCλ*^*m*567^) mutant into the Isl1:GFP background. *aPKCλ*^*m*567 ^mutants exhibit a mild posterior migration defect in which some FBMNs fail to migrate completely, remaining spread along the migratory pathway in r4 and r5 at 48 hpf (Figure 3B). In addition, *aPKCλ*^*m*567 ^mutants show a novel, partially penetrant phenotype (27%; n = 67) in which a subset of migrating FBMNs deviates from their normal path and mismigrates ventrally (Figure 3F, arrow; Table 1). As aPKCλ and aPKCζ have been shown to function redundantly in zebrafish retinal development, and are both present in the hindbrain [24], we hypothesized that they were functioning redundantly in this context as well. Single knockdown of either aPKCλ or aPKCζ using previously described morpholino oligonucleotides [24,25] followed by immunostaining with an antibody that recognizes both gene products [24], resulted in only a partial reduction in staining (data not shown). Simultaneous knockdown of both aPKCλ and aPKCζ, however, resulted in an absence of detectable aPKC staining in the hindbrain (Figure 3J). While injections of either single morpholino (MO) showed ventral mismigration defects similar in penetrance and severity to the *aPKCλ*^*m*567 ^mutant embryos (aPKCλ MO = 14%, n = 159; aPKCζ MO = 21%, n = 188), in aPKCλ+ζ double morphant embryos (or in *aPKCλ*^*m*567 ^mutants injected with aPKCζ MO) the penetrance of migration defects increased to 45% (n = 142) and the severity of defects (assayed by number of mismigrated cells and extent of migration) increased as well (Table 1; Figure 3C, G). In these double morphant embryos, mismigrated FBMNs form an ectopic ventral cluster that is elongated anteriorly, suggesting that FBMNs begin their posterior migration, mismigrate ventrally, then reverse their course and migrate anteriorly (Figure 3G, arrow). This ventral mismigration phenotype can be phenocopied in a dose-dependent manner by the injection of mRNA expressing a dominant negative form of aPKCλ [26] (Table 1).

**Table 1:** Summary of penetrance of mismigration phenotype in various conditions

Treatment	% ventral	n
250 μM aPKCλ MO	14	159
2 mM aPKCζ MO	21	188
250 μM aPKCλ MO + 2 mM aPKCζ MO	45	142
Uninjected	4	260
120 pg aPKCλ DN	16	45
300 pg aPKCλ DN	38	66
*aPKCλ*^*m*567/*m*567^	27	67
*aPKCλ*^*m*567/+ ^and +/+	3	192
*aPKCλ*^*m*567/*m*567 ^with 2 mM aPKCζ MO	41	22
*aPKCλ*^*m*567/+ ^or +/+ with 2 mM aPKCζ MO	15	95
*pard6gb*^*fh*266/*fh*266^	50	42
*pard6gb*^*fh*266/+ ^and +/+	3	156
2 mM Lama1 MO	88	16
1 mM Lama1 MO	60	86
*lamininα1*^*uw*1/*uw*1^	88	51

DN, Dominant Negative,; MO, morpholino.

We examined cross-sections of aPKCλ+ζ double morphant embryos stained with BODIPY TR methyl ester, a membrane dye [27]. This analysis revealed that the ectopic ventral clusters of FBMNs had escaped the hindbrain entirely, forming ectopia in mesenchyme ventral to the normal migratory path (Figure 3L, arrow). It is also clear from these sections that the architecture of the hindbrain is disrupted in aPKCλ+ζ double knockdown embryos. The cross-sectional area is smaller and rounder and the ventricle has failed to form, both expected outcomes of disrupting neural progenitor cell polarity (see Discussion).

**Figure 4:**
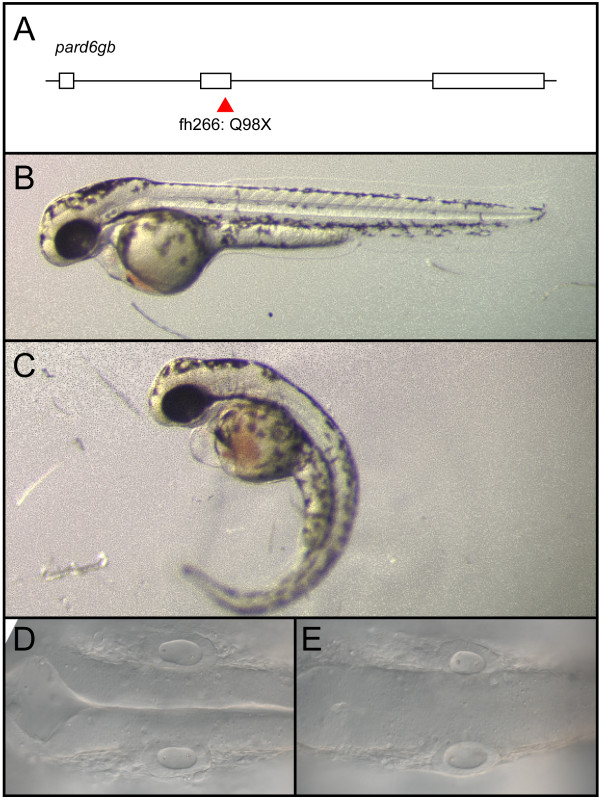
***pard6gb*^*fh*266 ^is a nonsense allele that results in edema, curved body axis, and a failure of the hindbrain ventricle to inflate**. **(A-E) **An N-ethyl-N-nitrosourea (ENU)-induced nonsense mutation at the end of exon 2 of *pard6gb*, *pard6gb*^*fh*266 ^(A) results in morphological defects at 48 hpf that include cardiac edema, a curved body axis (C), and a failure of the hindbrain ventricle to inflate (E) when compared to wild type (B, D). These defects are similar to those previously reported for a different allele of *pard6gb*.

To determine whether removal of other PAR-aPKC complex members resulted in a similar migration phenotype, we examined a *pard6gb *mutant generated by TILLING [28] that harbors a nonsense mutation in exon 2 (Figure 4A). We bred this allele into the *tg(isl1:GFP) *background and examined the incross for defects in FBMN migration. Incrosses produced *pard6gb*^*fh*266 ^homozygous mutant embryos with morphological defects identical to those reported for another *pard6gb *null allele (Figure 4) [11]. As in *aPKCλ*^*m*567 ^mutant and morphant embryos, FBMNs fail to migrate fully in *pard6gb*^*fh*266 ^homozygous mutant embryos, and a subset of *pard6gb*^*fh*266 ^mutant FBMNs mismigrate ventrally. This phenotype in *pard6gb*^*fh*266 ^mutants is less pronounced than in aPKC knockdown embryos, in that mismigrated FBMNs do not exit the hindbrain (Figure 3H).

### aPKC functions cell-nonautonomously to prevent ventral mismigration

**Figure 5:**
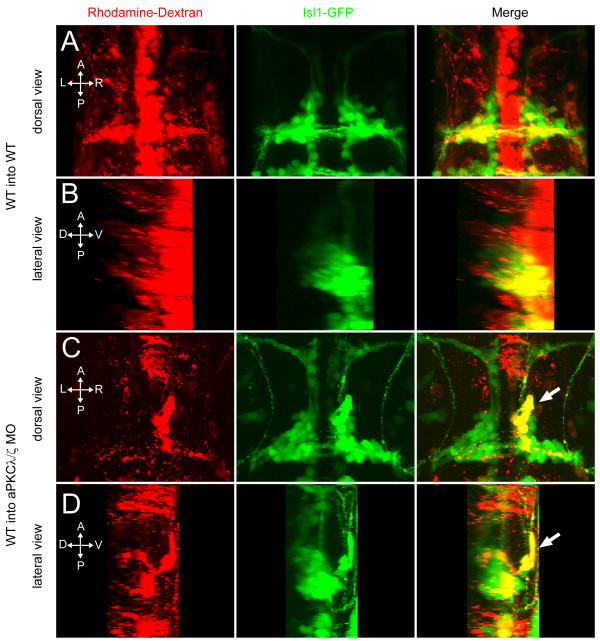
**aPKC functions cell non-autonomously to prevent ventral mismigration**. Maximum intensity projections of 48-hpf embryos in which rhodamine-dextran-labeled, *tg(isl1:GFP) *donor cells were transplanted into *tg(isl1:GFP) *transgenic hosts. **(A, B) **Yellow colocalization indicates donor-derived FBMNs. Wild-type (WT) donor cells transplanted into WT hosts never mismigrate ventrally. **(C, D) **A subset of WT FBMNs transplanted into an aPKCλ+ζ morphant host mismigrates ventrally (arrow).

In controlling FBMN migration, aPKC may function within the motor neurons, perhaps to determine their polarity during migration, or it may function in the surrounding neuroepithelium that lays down the appropriate boundaries, cues, and substrates for migration. In order to determine if aPKC is required within FBMNs themselves or within the surrounding tissue, we made mosaics by transplanting cells into the presumptive ventral hindbrain territory at the early gastrula stage [29,30]. When wild-type cells from *tg(isl1:GFP) *transgenic donors injected only with a rhodamine-tagged lineage dye are transplanted into uninjected *tg(isl1:GFP) *hosts, the donor-derived (rhodamine+; GFP+) FBMNs migrate along with their host-derived (rhodamine-; GFP+) counterparts to r6 and r7, and are never observed in an ectopic ventral position (Figure 5A, B). In contrast, when wild-type cells are transplanted into aPKCλ+ζ double morphant *tg(isl1:GFP) *hosts, a subset of both the host-derived FBMNs and donor-derived wild-type FBMNs mismigrates ventrally (Figure 5C, D). This behavior was observed in 10 out of 20 transplants in which donor-derived FBMNs were observed. This is a similar penetrance to that seen in untransplanted aPKCλ+ζ double morphants. This result shows that aPKC is required cell non-autonomously, in the environment of the migrating motor neurons. This non-autonomous function could be in other motor neurons, which serve as guides for donor-derived cells, or it could be in the surrounding neuroepithelium, which provides the substrate and directional cues for migration. We observe that mismigrated clusters of motor neurons can consist largely, if not exclusively, of wild-type donor-derived cells, suggesting that guidance cues from mismigrated host neurons are not required for donor neuron mismigration. Our further results below support a model in which neuroepithelial progenitor cell polarization is essential for normal FBMN migration.

### PAR-aPKC complex is required for maintenance of apical-basal polarity in the hindbrain neuroepithelium

Because our mosaic analysis showed that aPKC functions cell-nonautonomously with respect to FBMN migration, we examined the surrounding hindbrain tissue in PAR-aPKC complex mutants and morphants for defects that might explain the FBMN ventral mismigration defect. The earliest migrating FBMNs navigate through a hindbrain composed of neuroepithelial progenitor cells that are polarized apicobasally with their apical domains at the ventricular surface and basal domains at the pial surface. This polarity is reflected in apically localized centrosomes and cellular junctions and a basally localized basement membrane. It has been reported that in *aPKCλ*^*m*567 ^mutants, apical structures are formed correctly in neural epithelia (retina and neural tube) and are present during the time when the earliest-born FBMNs begin their migration [25]. These structures are not maintained, however, and therefore a loss of apical junctional markers ZO-1 and β-catenin is observed starting at about 40 hpf [25]. aPKCλ and aPKCζ have been shown to function redundantly in the retina [24], so we looked at markers of apical-basal polarity in aPKCλ+ζ double morphant embryos to determine if this polarity was disrupted in the hindbrain during the time when FBMNs are migrating.

**Figure 6:**
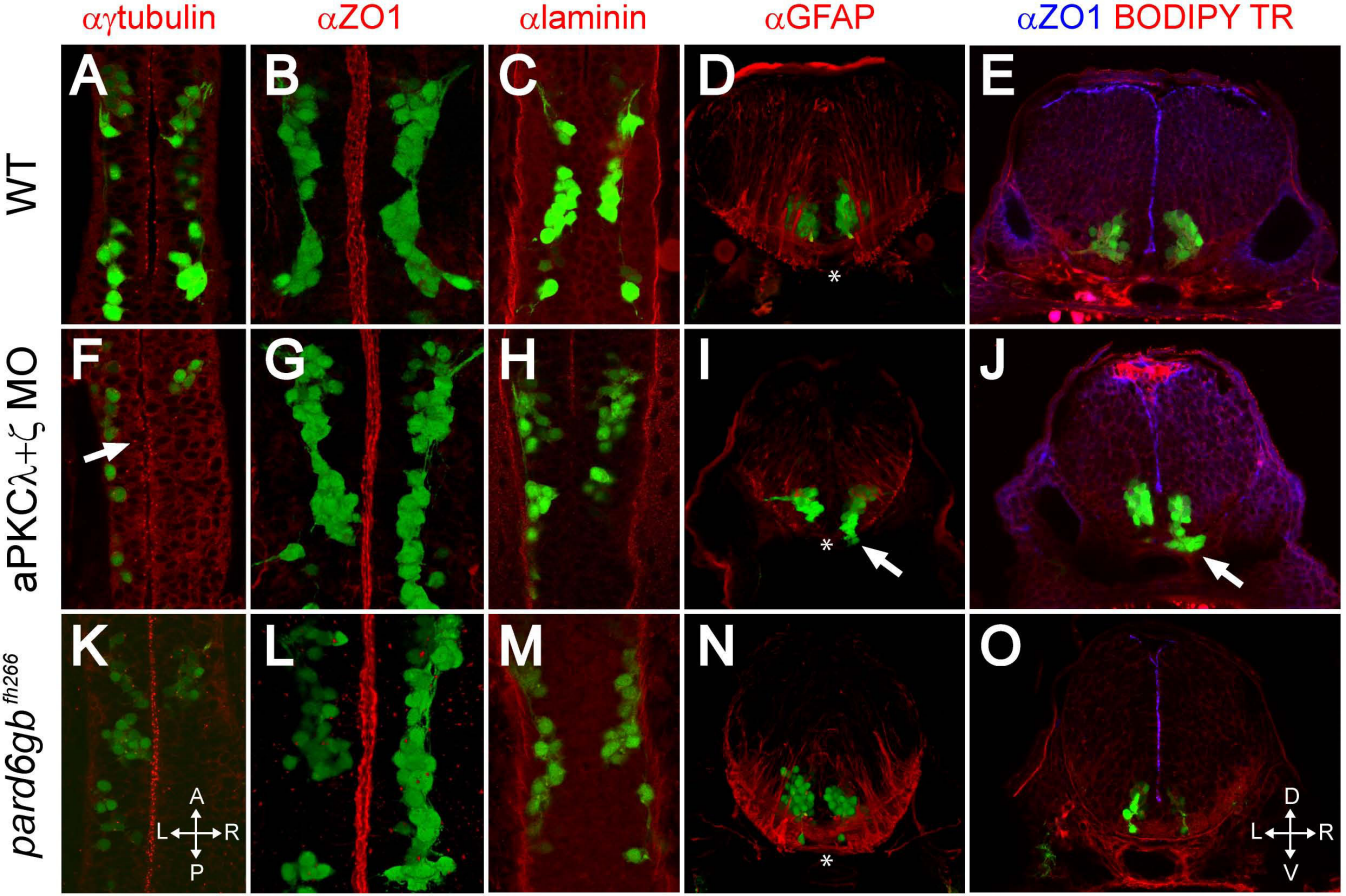
**Removal of aPKC or Pard6gb results in defects of apical midline formation and maintenance**. **(A-C, F-H, K-M) **Single XY sections (A, C, F, H, K, M) or three-dimensional reconstructions (B, G, L) of 24 hpf Isl1:GFP transgenic embryos stained for γ-tubulin (A), ZO1 (B), or Laminin (C) show that while apical tight junctions and basal basement membrane are formed correctly in the ventral hindbrain of embryos lacking aPKC or Pard6gb (B, C, G, H, L, M), there are subtle defects in neuroepithelial progenitor polarity reflected in misaligned centrosomes (F) (arrow). **(D, I, N) **Three-dimensional reconstructions of 70 μm thick 48-hpf cross-sections stained with α GFAP (α-Glial fibrillary acidic protein) show that while staining is reduced in aPKCλ+ζ double knockdown embryos, the absence of radial glial endfeet is unlikely to explain ventral mismigration as endfeet are absent from the most ventromedial region (where mismigration occurs in morphants, arrow) of both wild0type embryos and morphants (asterisks). **(E, J, O) **Single XY optical sections of 48-hpf vibratome cross-sections stained for ZO1 and with BODIPY TR methylester dye show reduced staining in aPKCλ+ζ double morphants in which ventral mismigration is occurring (J, arrow) or *pard6gb*^*fh*266 ^mutants (O), indicating that apical tight junctions are lost between 24 and 48 hpf in these embryos.

Focusing on the ventral hindbrain where FBMNs are migrating, we found that at 24 hpf in wild-type embryos, the centrosomes of neuroepithelial progenitor cells, visualized by anti-γ-tubulin staining, are localized apically, closely apposed to the ventricle, as is the junction protein ZO1 (Figure 6A, B). ZO1 is typically associated with tight junctions but it has been shown in mammals that tight junctions are disassembled during neurulation and ZO1 becomes associated with adherens junctions in the neuroepithelium [31] so this staining reflects the localization of whichever apical junctional complexes are present at this stage. In aPKCλ+ζ double morphants at 24 hpf, ZO1 staining is indistinguishable from that in wild type (Figure 6G, L), indicating that apical junctional complexes are formed even in the absence of any detectable aPKC. Anti-γ-tubulin staining of aPKCλ+ζ double morphants, however, reveals that while the majority of centrosomes are localized to the midline as in wild type, there is a small but significant increase in the number of centrosomes located more than 5 μm from the ventricular surface (11% (n = 642 centrosomes from 4 embryos) versus 6% in wild type (n = 633 centrosomes from 4 embryos); *t*-test, *P *< 0.05; Figure 6F, arrow). More severe midline defects have been reported in *pard6gb *mutants [11] and we also observe these defects in the dorsal hindbrain of *pard6gb*^*fh*266 ^mutant embryos but they are less dramatic in the ventral hindbrain where FBMN migration occurs (Figure 5K, L). The mislocalization of progenitor centrosomes in aPKCλ+ζ double morphants and *pard6gb *mutants suggests that while apical-basal polarity is not abolished, there is a defect in progenitor cells' ability to localize their subcellular components and align apicobasally that results in a defect in neural tube architecture.

Staining of coronal vibratome sections through wild-type 48-hpf embryos at the level of hindbrain rhomobomere 4/5 shows that ZO1 is present at the ventricular surface at 48 hpf (Figure 6E). In both aPKCλ+ζ double morphant and pard6γ b mutant embryos, ZO1 staining is discontinuous and reduced (Figure 6E, J, O), although the amount of staining lost is highly variable. It has been shown that in aPKCλ mutants apical polarity is established correctly but apical structures are gradually lost starting at about 40 hpf in the spinal cord [25,32]. Our data are consistent with the idea that the PAR-aPKC complex is required for the maintenance of apical structures but that apical-basal polarity is not completely lost in embryos in which aPKC or Pard6gb is removed. These observations are also consistent with the timing of onset of FBMN ventral mismigration as migration in both aPKCλ+ζ double morphant embryos and *pard6gb*^*fh*266 ^mutant embryos is normal at 24 hpf (Figure 6A-C, F-H, K-M) while ventral ectopias are observed at 48 hpf (Figure 6I, J, arrows).

Roberts and Appel [32] reported a reduction of radial glia at 72 hpf in the spinal cord of aPKCλ mutants. We wondered if a disruption of basal glial endfeet could explain the ventral mismigration we observed in aPKCλ+ζ double morphant embryos. Staining for GFAP (Glial fibrillary acidic protein), a glial marker, reveals that basal glial endfeet are formed properly in aPKCλ+ζ double morphants and *pard6gb*^*fh*266 ^mutants (Figure 6D, I, N), although the overall architecture and number of radial glia is disrupted due to the altered morphology of the hindbrain. Escaping FBMNs in aPKCλ+ζ double morphant embryos are always located ventro-medially in a region where glial endfeet are absent in both wild type and double morphants (Figure 6D, I, N, asterisk), suggesting that glial endfeet are not responsible for preventing mismigration. The most ventro-medial tissue in these sections is the floorplate, which is also properly formed in aPKCλ+ζ double morphants as seen by Zn5 staining (data not shown).

### FBMNs escape the hindbrain through holes in Laminin

**Figure 7:**
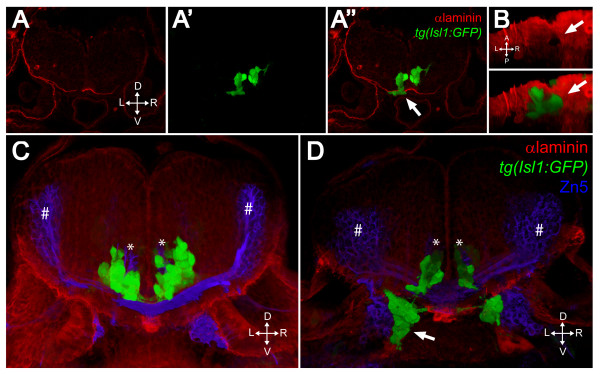
**FBMNs exit the hindbrain through holes in the ventral Laminin-containing basement membrane that can be phenocopied by Laminin knockdown**. **(A-A") **Single XY section of a vibratome cross-section through a 48-hpf aPKCλ+ζ, *tg(isl1:GFP) *double morphant stained for Laminin (red). Ventrally mismigrating FBMNs exit the hindbrain through a hole in Laminin (arrow). **(B) **Ventral view of a three-dimensional reconstruction of the same coronal section showing the Laminin hole and the mismigrating FBMNs (arrows). **(C, D) **Three-dimensional reconstructions of 70 μm thick vibratome cross-sections through 48- hpf wild-type (C) and Lamininα 1 morpholino-injected *tg(isl1:GFP) *embryos (D) stained for Laminin (red) and Zn5 (blue) showing that Laminin knockdown results in ventrally mismigrated FBMNs (arrow) that exit the hindbrain while leaving abducens motor neurons (asterisks) and commissural interneurons (hash symbols) unaffected. ZN5-staining neurons outside the hindbrain are the sensory neurons of the acoustic nerve (nVIII).

In spite of relatively subtle defects in apicobasal polarity in the ventral hindbrain neuroepithelium, migrating FBMNs mismigrate ventrally, frequently escaping the hindbrain altogether. Laminin staining appears normal in whole-mount aPKCλ+ζ double morphants and *pard6gb *mutants at 24 hpf, indicating that the basal domain is established correctly in these embryos (Figure 6C, H, M). However, by 48 hpf, holes are detected in the Laminin-containing basement membrane through which FBMNs have escaped. A single XY section shows such a discontinuity in the Laminin staining (Figure 7A) while a three-dimensional reconstruction of the same embryo shows that the motor neurons have escaped from a hole of about the size of one cell diameter and then migrated around it outside the ventral surface of the hindbrain (Figure 7B; Additional file 3). These holes occur at various anterior to posterior levels and are never seen in wild-type embryos (data not shown). They do not correspond with the exit points of any of the axons of the *isl1:GFP *positive branchiomotor nerves or of the abducens motor nerve (data not shown) and are not bilaterally symmetrical, suggesting that their location is likely random.

### Ventral mismigration can be phenocopied by Laminin depletion

If ventral mismigration seen in aPKC knockdown is due to a defect in the Laminin-containing ventral basement membrane, we would expect to see similar mismigration if Laminin levels in the basement membrane are reduced. It has been reported that *bashful/lamininα1 *mutants have a defect in the posterior migration of FBMNs [33] but the nature of that defect was unclear. In addition, genetic interactions between *lama1 *and other pathways implicated in FBMN migration have been reported [34]. Cross-sections through either *lama1 *mutant (*bal*^*uw*1^) or *lama1 *MO 48-hpf embryos stained with a polyclonal Laminin antibody reveal that while some Laminin staining is preserved at the midline, there are large swaths of Laminin missing ventrally through which large numbers of FBMNs escape, forming large motor neuron ectopia outside the hindbrain (Figure 7C, D, arrow). This phenotype is seen at a higher penetrance than in aPKC double morphant embryos (Table 1) with more FBMNs contributing to these ectopic clusters. Laminin depletion, then, results in basement membrane discontinuities and ventral mismigration of FBMNs similar to that seen upon PAR-aPKC complex depletion. We conclude that the ventral mismigration observed in aPKCλ+ζ double morphant embryos is due to a loss of integrity of the Laminin-containing basement membrane.

### In the absence of Laminin, FBMNs fail to speed up and reorient their centrosomes

**Figure 8:**
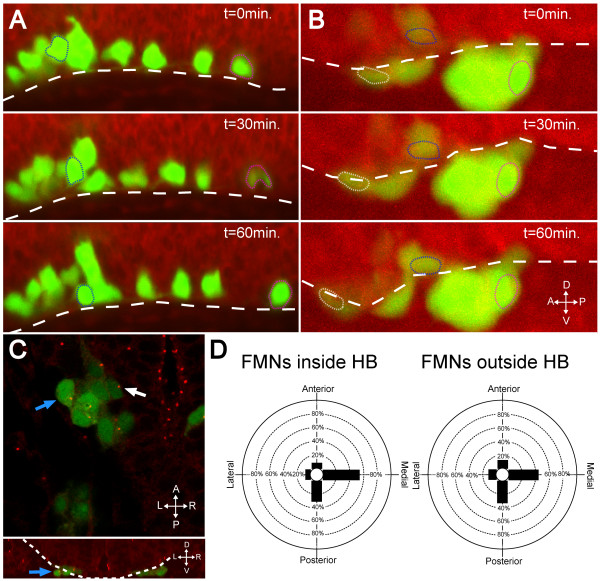
**In the absence of Laminin, FBMNs fail to speed up and do not reorient centrosomes**. **(A, B) **Maximum intensity projections from timelapse recordings started at 24 hpf of wild-type (A) and *lamininα1*-/- (B) *tg(isl1:GFP) *(green) embryos stained with BODIPY TR methyl ester dye (red) reveal that FBMNs migrate ventrally down to the ventral limit of the hindbrain (dotted line) in both the presence and absence of Laminin (compare blue outlined FBMNs), they do not speed up in the absence of Laminin (compare magenta outlined FBMNs) and some even mismigrate anteriorly (white outlined FBMN). **(C) ***lamininα1*-/-; *tg(isl1:GFP) *embryos stained for γ-tubulin show centrosomes pointing medially in FBMNs both inside the hindbrain (white arrow) and outside the hindbrain (blue arrows). The upper panel is an XY section while lower panel is a reconstructed YZ section at the level of the FBMN indicated by the blue arrow in the upper panel with the hindbrain outlined (dotted line) to show that the indicated FBMN is outside the hindbrain. **(D) **Quantification of centrosome positioning in *lamininα1*-/- embryos shows that centrosomes fail to reorient in the absence of Laminin (compare to Figure 2D). HB, hindbrain.

If the Laminin-containing basement membrane is a substrate for migration that serves to induce the changes in velocity and centrosome orientation that we have observed in wild-type embryos, then we would expect those changes not to occur in the absence of Laminin. In order to test this, we performed live imaging of wild-type and *lamininα-/-tg(isl1:GFP) *embryos. Counterstaining with BODIPY TR methyl ester dye and imaging in a lateral view allowed us to visualize ventral and posterior migration with respect to the ventral surface of the hindbrain (Figure 8A, B; Additional files 4 and 5). We observed that in both the presence and absence of Laminin, FBMNs undergo ventral migration down to the ventral surface of the hindbrain (Figure 8A, B; Additional files 4 and 5, blue outline). In wild-type embryos we observed that cells that were in contact with the ventral surface of the hindbrain move quickly posteriorly as described in Figure 1 (Figure 8A; Additional file 4, magenta outline). In *lamininα1*-/- embryos, however, FBMNs continue their ventral migration and exit the hindbrain. Once they are outside the hindbrain they fail to speed up posteriorly (Figure 8B; Additional file 5, magenta outline) and, in some cases, even mismigrate anteriorly (Figure 8B, white outline).

To test whether FBMNs reorient their centrosomes in the absence of Laminin, we stained *lamininα1*-/-; *tg(isl1:GFP) *embryos for γ-tubulin and compared the orientation of centrosomes in FBMNs that were inside the hindbrain with those that were outside the hindbrain. Most centrosomes in both FBMNs inside and outside the hindbrain were oriented medially in a distribution that was the same as that seen for wild-type centrosomes that were dorsal to the Laminin-containing basement membrane (compare Figures 2D and 8D). By chi-squared analysis, the distribution of centrosomes inside the hindbrain was not significantly different from that of centrosomes outside the hindbrain (*P *> 0.2). This suggests that in the absence of Laminin, FBMNs fail to reorient their centrosomes and maintain the polarity of FBMNs in the ventral phase of migration. Together, these data show that the faster, posterior-directed phase of migration with centrosomes oriented along the anterior-posterior axis is dependent on the presence of an intact Laminin-containing basement membrane.

## Discussion

We show here that FBMNs initially migrate ventrally until they come into contact with the Laminin-containing basement membrane, which appears to serve as a substrate for migration. Upon contact with the basement membrane, FBMNs undergo a change in velocity and centrosome orientation. Knockdown of aPKCλ and aPKCζ results in disruption of the basement membrane and a mismigration of FBMNs in which they exit the hindbrain through holes in basal Laminin. This mismigration can be phenocopied by mutation (or knockdown) of Lamininα 1, suggesting that it is the disruption of the basement membrane that is responsible for ventral mismigration. It must be noted, however, that in *pard6gb*^*fh*266 ^embryos FBMNs mismigrate ventrally but do not exit the hindbrain, indicating that mismigration can occur without a breach in the integrity of the basement membrane. It is likely, then, that there are other basal structures disrupted in the *pard6gb*^*fh*266 ^hindbrain that also serve to prevent ventral mismigration.

What is the nature, then, of the defects in the hindbrain that result in ventral mismigration? Depletion of members of the PAR-aPKC complex aPKCλ, aPKCζ, or Pard6gb results in a disruption in the architecture of the hindbrain resulting in a smaller cross-sectional area and a failure of the ventricle to inflate. Two mechanisms are likely at work here to produce such a phenotype. Munson *et al*. [11] report a similar ventricle defect in *pard6gb*^*s*441 ^mutants, due to a failure to correctly establish apical identity in neural progenitors. In addition, Roberts and Appel [32] report that loss of apical-basal polarity in *aPKCλ*^*m*567 ^mutants results in spindle orientation defects in progenitor cells and premature differentiation. The defects in hindbrain architecture we observe, then, are likely due to a combination of morphogenesis and differentiation defects. We conclude that these defects result, directly or indirectly, in a disruption of the basal structures of the hindbrain, including the basement membrane, that result in ventral mismigration of FBMNs.

### The Laminin-containing basement membrane as substrate for migration

The observation that FBMNs reorient their centrosomes and accelerate their posterior migration upon contact with the Laminin-containing basement membrane suggests that the basement membrane is a substrate for migration that allows for more efficient posterior-directed motion. In the absence of Laminin, FBMNs fail to speed up and reorient their centrosomes, suggesting that Laminin is required for the changes in velocity and centrosome reorientation. It remains unclear whether this substrate actively directs migration or simply provides a permissive milieu that allows for faster migration. Signaling between Laminin and Integrin receptors has been shown to be important in a number of neuronal migrations, such as the anterior migration of neuroblasts to the olfactory bulb [35], and the radial migration that forms the layers of the mammalian cortex [36]. Furthermore, Laminin may be involved directly in the signaling pathways that are required for FBMN migration. Sittaramane *et al*. [34] showed pairwise genetic interactions between Lamininα 1, the cell adhesion molecule Tag1, and the transmembrane PCP protein Strabismus in the regulation of FBMN migration, suggesting that there is a direct interaction between Laminin and the adhesion and polarity molecules that control directional migration; however, the molecular mechanism for that interaction is unknown. Both Paulus *et al*. [33] and Sittaramane *et al*. [34] described the defect in *lama1 *mutants as a defect in posterior migration and not, as we have described here, as a ventral mismigration. Failure to migrate posteriorly is likely to be a consequence of ventral mismigration and escape from the hindbrain but that does not preclude a role for Laminin as either a permissive or instructive substrate for migration.

### The Laminin-containing basement membrane as a boundary that prevents ventral mismigration

Much of the previous work on FBMN migration has focused on the role of the PCP pathway. It is clear from the work of many labs that genes in this pathway are required for the migration of FBMNs [4-7,37] but the mechanism for this requirement is unclear. Wada and colleagues [7] observed that in *fz3a *and *celsr2 *mutants, FBMNs fail to migrate posteriorly and instead mismigrate dorsally within r4. This observation, along with evidence from genetic mosaics, led them to posit a model in which the PCP pathway is required cell-nonautonomously to restrict FBMN migration to the ventral pial surface [7,8].

Our analysis of the wild-type behavior of migrating FBMNs is consistent with the idea that the dorsal-ventral position of FBMNs is important for their migration. FBMNs begin their migration traveling ventro-posteriorly but any further ventral migration is blocked by contact with the ventral basement membrane. This initial ventral migration may indeed be due to an exclusion from the more dorsal neuroepithelial progenitor cells as proposed by Wada *et al*. [7] or there may be other guidance cues directing FBMNs ventrally. The basement membrane, then, functions as a complementary ventral boundary to the dorsal boundary provided by PCP components, preventing further ventral migration and keeping the migratory pathway sandwiched at an exact dorsal-ventral level. The picture that arises, then, is one in which the dorsal-ventral level of the FBMN migratory pathway is tightly defined by the opposing actions of a signaling pathway on the dorsal side and a physical boundary (that also serves as a substrate for migration) on the ventral side.

### FBMN ectopia resembles cobblestone lissencephaly

Cobblestone lissencephaly is a brain malformation found in a number of clinical syndromes, including Walker-Warburg syndrome (WWS), muscle-eye-brain disease and Fukuyama congenital muscular dystrophy. It is characterized by cortical overmigration in which radially migrating cortical neurons migrate past the pial basement membrane, resulting in a cobblestone-like texture in the cortex [38]. Walker-Warburg syndrome and Fukuyama congenital muscular dystrophy have been linked with hypoglycosylation of α-Dystroglycan, which is normally required for Dystroglycan-Laminin binding [39,40]. In addition, mouse mutations that disrupt cell-basement membrane interactions or basement membrane integrity, such as tissue-specific deletion of integrin-linked kinase or focal adhesion kinase, result in phenotypes that are similar to clinical cobblestone lissencephalies [41,42]. These defects all show that disruption of the cortical pial basal lamina that serves as the boundary to radial migration results in ectopic migration in which neurons escape the brain.

These cobblestone lissencephaly phenotypes are similar to the motor neuron ectopia described here; however, the underlying genetic causes are different. Whereas mammalian cortical neuron ectopia are caused by the loss of proteins that function directly in the establishment of, or signaling by, the cortical basement membrane, the motor neuron ectopia we describe are due to the loss of PAR-aPKC complex polarity determinants functioning in the environment of the motor neurons. However, we observed discontinuities in the neuroepithelial basement membrane in aPKC mutants and morphants through which FBMNs appear to escape, and these defects were phenocopied by direct knockdown of Laminin itself. We hypothesize that subtle defects in the polarization of neuroepithelial progenitor cells in aPKC mutants causes reduced basal deposition or anchoring of Laminin-containing basement membrane, resulting in holes or weaknesses through which FBMNs escape the hindbrain. It is clear, however, that other models are possible. In addition to midline polarity defects we also observed a decrease in the size of the hindbrain, which is attributable to loss of progenitors as seen by Roberts and Appel [32]. This premature loss of progenitors with neuroepithelial character could result in regions of the pial surface that are lacking a basal surface on which to anchor Laminin polymerization, resulting in the escape of FBMNs.

## Conclusions

We have shown that facial branchiomotor neurons undergo a change in velocity and polarity upon contact with the ventral Laminin-containing basement membrane. Disruption of PAR-aPKC complex members results in defects in the basal structures of the neuroepithelium, including the basement membrane, that result in ectopic ventral migration of FBMNs and their escape from the hindbrain. We conclude that the Laminin-containing ventral basement membrane, dependent on the action of the apical PAR-aPKC complex, is both a substrate for migration and a boundary that constrains facial motor neurons to the appropriate migratory path.

## Methods

### Animals

Zebrafish (*Danio rerio*) were maintained as described [43]. *tg(isl1:GFP) *[20] were used as wild-type controls and crossed into *has*^*m*567 ^[25] and *bal*^*uw*1 ^[33] and *pard6gb*^*fh*266 ^mutant backgrounds. Adults and embryos were genotyped by PCR amplification of a region flanking the mutation followed by restriction digest (*has*^*m*567^, AGGAACGGCTGGGATGTC and TCTCGAGTCAAAACATCCAAGA, Bfi1 cuts wild-type sequence; *pard6gb*^*fh*266^, CCATTCCTGCAGTGTTGGCAAATTC and  AACAGCACAAGGCAAAACTGGGTCA, BsmA1 cuts wild-type sequence), or by single pair matings followed by morphological identification of mutants (*bal*^*uw*1^). Embryos were raised at 28.5°C. In some cases pigment development was inhibited by the use of phenyl thiourea as described in Westerfield [43]. All of the animal protocols for use of zebrafish in the research described in this paper are in compliance with internationally recognized guidelines for the use of fish in biomedical research. The protocols were approved by the Fred Hutchinson Cancer Research Center Institutional Animal Care and Use Committee, file #1392 'The Genetic Control of Hindbrain Patterning in the Zebrafish', Cecilia Moens PI.

### Construction of mutant and transgenic lines

#### tg(zCREST1:memb-mRFP1)

Briefly, the zCREST1 enhancer element [18] was PCR amplified from the Isl1:GFP construct [20] with primers that added restriction sites, subcloned through β-Globin-GFP [44] and into pHSP70 [45] upstream of the HSP70 promoter. memb-mRFP1 [17] was subcloned into zCREST1-HSP70 downstream of the HSP70 promoter. Plasmid DNA was dialyzed against distilled water and microinjected into early one-cell embryos at 50 ng/μl, transient transgenics were raised, and one stably integrated line retained.

#### tg(krox20BAC:GFP)

Briefly, a PCR product was generated that created a GFP-Kan fusion with 48 bp of *krox-20/egr2b *on each end that are approximately 1,000 bp apart in the *krox-20 *coding region. This product was recombined into zebrafish BAC CH211-78A5 using the ET recombination system [46], creating a fusion gene containing the first ten codons of *krox-20 *fused in frame to GFP, driven by the endogenous *krox-20 *promoter region.

#### pard6gb^fh266^

A mutant allele of *pard6gb *was identified by TILLING [28]. The allele contains a nonsense mutation in exon 2 (Q98X) that truncates the protein amino-terminal to its known PDZ protein-protein interaction domain (Figure 4).

### Live confocal imaging

*Tg(zCREST1:memb-mRFP1); Tg(krox20BAC:GFP) *double transgenic embryos at 18 to 24 hpf were embedded in 1.2% agarose in embryo medium in glass bottom dishes (MatTek, Ashland, MA, USA) oriented with their hindbrain against the glass. They were imaged on a Zeiss Pascal inverted confocal microscope using a water immersion 40× objective. Single FBMNs were tracked in Z and time, allowing us to measure, at each timepoint, the distance between the posterior-most extent of a cell's cytoplasm (as determined by the inner edge of membrane fluorescence) and the r4-r5 boundary (determined by *krox20:GFP *fluorescence in r5). Measurements were made using Zeiss LSM software. Extent of migration (μm) was plotted against time (minutes) and the plots were analyzed for the fit of a bilinear model as described [19].

### Morpholino, mRNA, and dye injections

Zebrafish embryos at the one-cell stage were injected with 1 nl of MO, mRNA, or fixable rhodamine-dextran (Invitrogen, Carlsbad, CA, USA). aPKCλ MO (250 μM) [25] and aPKCζ MO (2 mM) [24] were obtained from GeneTools (Philomath, OR, USA). Lama1 MO (1 mM; MO1) [47] was a gift from Anand Chandrasekhar. pCS2+aPKCi-AA (aPKCλ dominant negative) [26] was a gift from Salim Abdelilah-Seyfried and was used to make capped mRNA with the mMESSAGE mMACHINE kit (Ambion, Austin TX, USA).

### Sectioning and immunohystochemistry

Embryos were fixed in 4% paraformaldehyde overnight at 4°C and either stained in wholemount or equilibrated in 0.3 M sucrose, mounted in 17% gelatin and sectioned to 70 μm using a vibratome followed by staining. Staining was performed as described [48] except -20°C acetone was used for permeabilization and Roche Western blocking reagent was used for blocking (Roche, Basel Switzerland). Primary antibodies used were: Laminin AB-1 (NeoMarkers, Fremont, CA, USA; 1:200), EphA4 (Upstate, Lake Placid, NY, USA; 1:200), γ-Tubulin (Sigma, St. Louis, MO, USA; 1:2500), aPKC (Santa Cruz C20, (Santa Cruz, CA, USA; 1:1,000), ZO1 (Zymed, San Francisco, CA, USA; 1:1,000), GFAP (Glial fibrillary acidic protein; Dako, Glostrup, Denmark; 1:200), acetylated Tubulin (Sigma; 1:2,000). Secondary antibodies to mouse, rabbit, and goat conjugated with AlexaFluor 405, 488, and 594 used at 1:200 (Invitrogen). When used, BODIPY TR methyl ester dye (Molecular Probes) was added to secondary antibody staining solution at 1:100. Tissue was dehydrated in glycerol then mounted in SlowFade Gold (Invitrogen). Imaging was as above. Three-dimensional reconstructions were performed using 'Three-dimensional opacity' mode in the Volocity software package (Improvision, Boston, MA, USA).

## Abbreviations

FBMN: facial branchiomotor neuron; GFP: green fluorescent protein; hpf: hours post-fertilization; MO: morpholino; PCP: planar cell polarity; r: rhombomere.

## Competing interests

The authors declare that they have no competing interests.

## Authors' contributions

PKG conceived of and carried out the experiments and drafted the manuscript. CBM conceived of the study, and participated in its design and coordination and helped to draft the manuscript. All authors read and approved the final manuscript.

## Supplementary Material

Additional file 1 **Timelapse movie of *Tg(zCREST1:memb-mRFP1)***. The movie was started at 18 hpf. Images were taken every 6 minutes. The movie runs at 4 frames per second. The embryo is shown in dorsal view. Click here for file

Additional file 2 **Timelapse movie of *tg(zCREST1:memb-mRFP1); tg(krox20BAC:GFP) *double transgenic**. The movie was started at 24 hpf. Images were taken every 6 minutes. The movie runs at 7 frames per second. The embryo is shown in dorsal view. Click here for file

Additional file 3 **Rotation of a three-dimensional reconstruction of the embryo shown in Figure 7A, B**. aPKCλ+ζ double MO *tg(isl1:GFP) *(green) 48-hpf embryo immunostained for Laminin (red). Cross-section. Click here for file

Additional file 4 **Timelapse movie of wild-type *tg(isl1:GFP) *embryo counterstained with BODIPY TR methyl ester dye**. The movie was started at 24 hpf. Images were taken every 5 minutes. The movie runs at 2 frames per second. The embryo is shown in lateral view. White line delineates the ventral limit of the hindbrain. The blue outlined cell moves ventrally while the magenta outlined cell moves posteriorly. Click here for file

Additional file 5 **Timelapse movie of *lamininα1*-/-; *tg(isl1:GFP) *embryo counterstained with BODIPY TR methyl ester dye**. The movie was started at 24 hpf. Images were taken every 6 minutes. The movie runs at 2 frames per second. The embryo is shown in lateral view. The white line delineates the ventral limit of the hindbrain. The blue outlined cell moves ventrally while the magenta outlined cell fails to move posteriorly and the white outlined cell moves anteriorly. Click here for file
